# A Staff Question at Cardiff

**Published:** 1912-12-21

**Authors:** 


### A Staff Question at Cardiff.

### CHANGES IN ELECTION TO THE HONORARY STAFF.

At the monthly meeting of the board of management of King Edward VII.'s Hospital, Cardiff, Colonel Bruce Vaughan moved a resolution that a committee be appointed to consider and report on the advisability of altering the present rules governing the election of members of the honorary medical staff. It appeared from Colonel Bruce Vaughan's remarks that every person who contributed at least ten guineas a year was entitled to attend and vote at the meetings of the board of management, and that any body of workmen contributing a similar amount were entitled to have a representative on the board. As the elections to the honorary medical staff are made by the board of management, it followed that some 400 people were entitled to vote at such elections, many of whom have only a slight interest in the actual work of the hospital, and rarely, and perhaps never, come near it, except to vote for some candidate who has been energetic enough to canvass them or who may by some chance be known to them personally. Colonel Vaughan contended that, however well the system had served its purpose in times gone by, it is quite unsuited to one of the best modern hospitals in the provinces and to the medical school, the completion of which only awaits the opening of the remaining fifty beds in the new wing.

Without proposing to discuss the new election scheme at that meeting, Colonel Vaughan expressed the wish that it will take the form of officers rather than persons—for instance, the chairmen of the standing committees, together with the Principal of the College and the Dean of the Medical Faculty, and a reasonable number of members of the board of management, elected for this special purpose. The proposal was seconded by Mr. Isaac Samuel, and powerfully supported by the chairman of the board of management (Major-General Lee), Dr. Herbert Vachell, and Principal Griffiths. The resolution was carried unanimously by a large meeting, representative of every section of the board, including the colliery and working men contributors, and a committee was accordingly appointed to submit the details of the proposed scheme to a future meeting. The unanimity with which Colonel Vaughan's remarks was received is held to augur well for a happy issue from the present unsatisfactory method.

